# The Active Compounds and Therapeutic Target of *Tripterygium wilfordii Hook. f*. in Attenuating Proteinuria in Diabetic Nephropathy: A Review

**DOI:** 10.3389/fmed.2021.747922

**Published:** 2021-09-21

**Authors:** Peng Liu, Jing Zhang, Yun Wang, Zhengri Shen, Chen Wang, Dan-Qian Chen, Xinping Qiu

**Affiliations:** ^1^Shunyi Hospital, Beijing Hospital of Traditional Chinese Medicine, Beijing, China; ^2^Institute of Plant Resources, Yunnan University, Kunming, China; ^3^Department of Emergency, China-Japan Friendship Hospital, Beijing, China

**Keywords:** diabetic nephropathy, *Tripterygium wilfordii Hook f*., tripterygium wilfordii polyglycosides, triptolide, celastrol

## Abstract

*Tripterygium wilfordii Hook. f*. (TWHF) is a traditional Chinese herbal medicine and widely used to treat diabetic kidney disease in China. Emerging evidences have revealed its ability to attenuate diabetic nephropathy (DN). Tripterygium wilfordii polyglycosides (TWPs), triptolide (TP), and celastrol are predominantly active compounds isolated from TWHF. The effects and molecular mechanisms of TWHF and its active compounds have been investigated in recent years. Currently, it is becoming clearer that the effects of TWHF and its active compounds involve in anti-inflammation, anti-oxidative stress, anti-fibrosis, regulating autophagy, apoptosis, and protecting podocytes effect. This review presents an overview of the current findings related to the effects and mechanisms of TWHF and its active compounds in therapies of DN, thus providing a systematic understanding of the mechanisms and therapeutic targets by which TWHF and its active compounds affect cells and tissues *in vitro* and *in vivo*.

## Introduction

Diabetic nephropathy (DN) is defined as decreased renal function with persistent clinically detectable proteinuria (1). As a serious microvascular complication of types 1 or 2 diabetes mellitus (DM), DN occurs in ~25–40% of patients with DM, and has become the leading cause of end-stage renal disease (ESRD) in China (2, 3). Approximately 463 million people suffers from DM worldwide in 2019, and are expected to raise up to 700 million untill 2045 (4).

Proteinuria, an independent risk factor of disease progression, is the most important clinical characteristic of DN. The presence of microalbuminuria can increase all-cause mortality in patients with diabetes mellitus (DM) (5). Without early intervention, ~50% of DM patients with microalbuminuria will progress to macroalbuminuria (6, 7). Although several recent studies have confirmed that angiotensin-converting enzyme inhibitors (ACEIs)/angiotensin receptor blockers (ARBs) can reduce DN proteinuria and delay disease progression (8, 9), these have been shown to be ineffective in DN patients with normal blood pressure (10).

Various traditional Chinese herbal medicine (CHM) has been shown to be effective in the treatment of proteinuria (11, 12). *Tripterygium wilfordii Hook. f*. (TWHF), also known as Lei Gong Teng, is a traditional CHM which is widely used in the treatment of the inflammation and autoimmune disorders (13–15). Based on its diverse pharmacological activities, TWHF has been used to treat different diseases, such as cancer, rheumatoid arthritis, and Crohn's disease (16–18). Recent experimental and clinical studies have demonstrated that TWHF could significantly reduce proteinuria, protect renal function, and attenuate kidney injury (19–21).

Several randomized controlled clinical trials have found that TWHF possibly imparts nephroprotective effects by decreasing proteinuria, serum creatinine (Scr) levels, and blood urea nitrogen (BUN) levels (22–24). A network pharmacology research showed that TWHF may play a role in treating DN through AGE-RAGE signaling pathway, TNF signaling pathway, IL-17 signaling pathway, insulin resistance, and calcium signaling pathway (25). However, the underlying mechanisms by which TWHF and its active compounds attenuate proteinuria in DN remain unclear. This review discusses the molecular mechanisms of TWHF therapies in proteinuria in DN.

## Main Active Compounds of TWHF

TWHF belongs to genus Tripterygium of family celastraceous, and its main bioactive ingredients include terpenoids, tripterygium wilfordii polyglycosides (TWPs), lignans, glycosides, and alkaloids. The terpenoids of TWHF are constituted by sesquiterpenes, diterpenes (triptonide, tripdiolide, and triptolide), triterpenes (wilforlide A, pristimerin, and celastrol) (26, 27).

**Figure 1:**
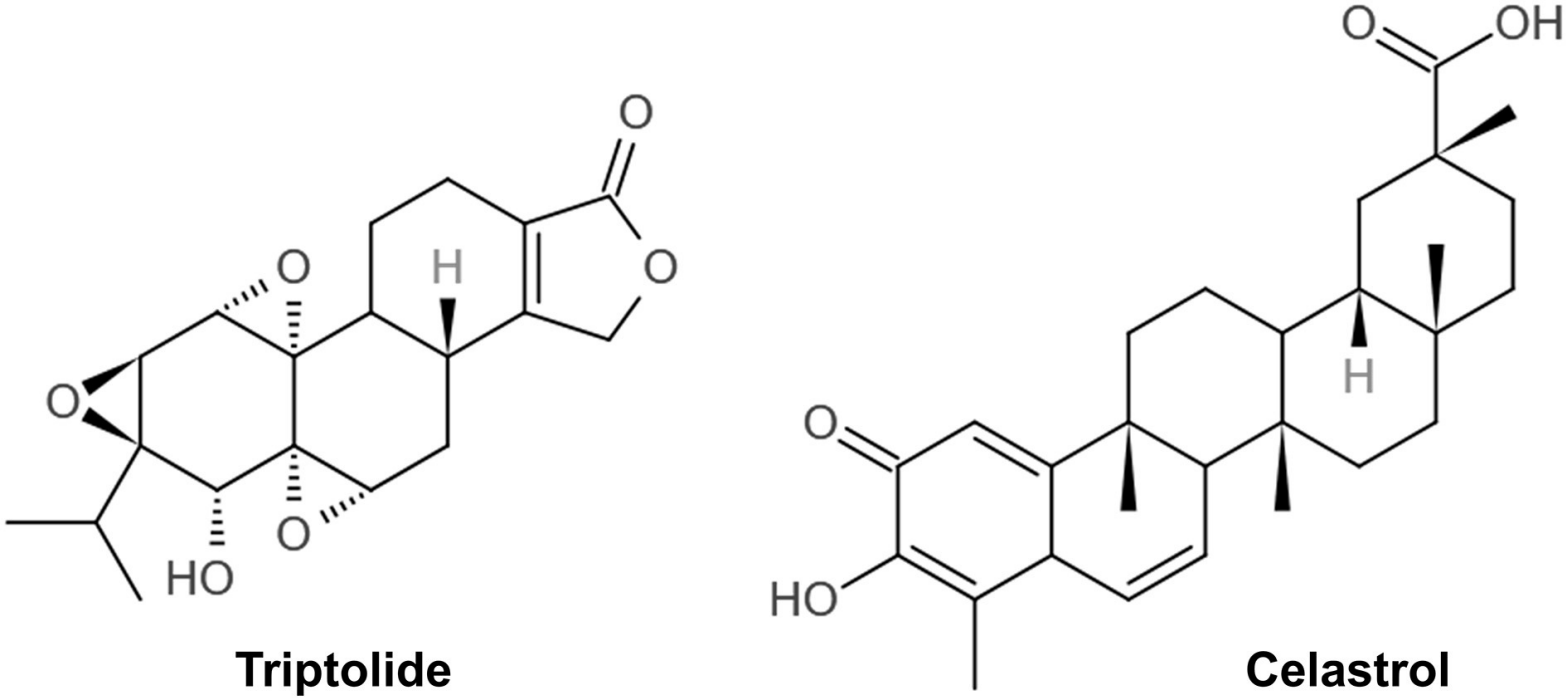
The chemical structure of triptolide and celastrol.

**Figure 2:**
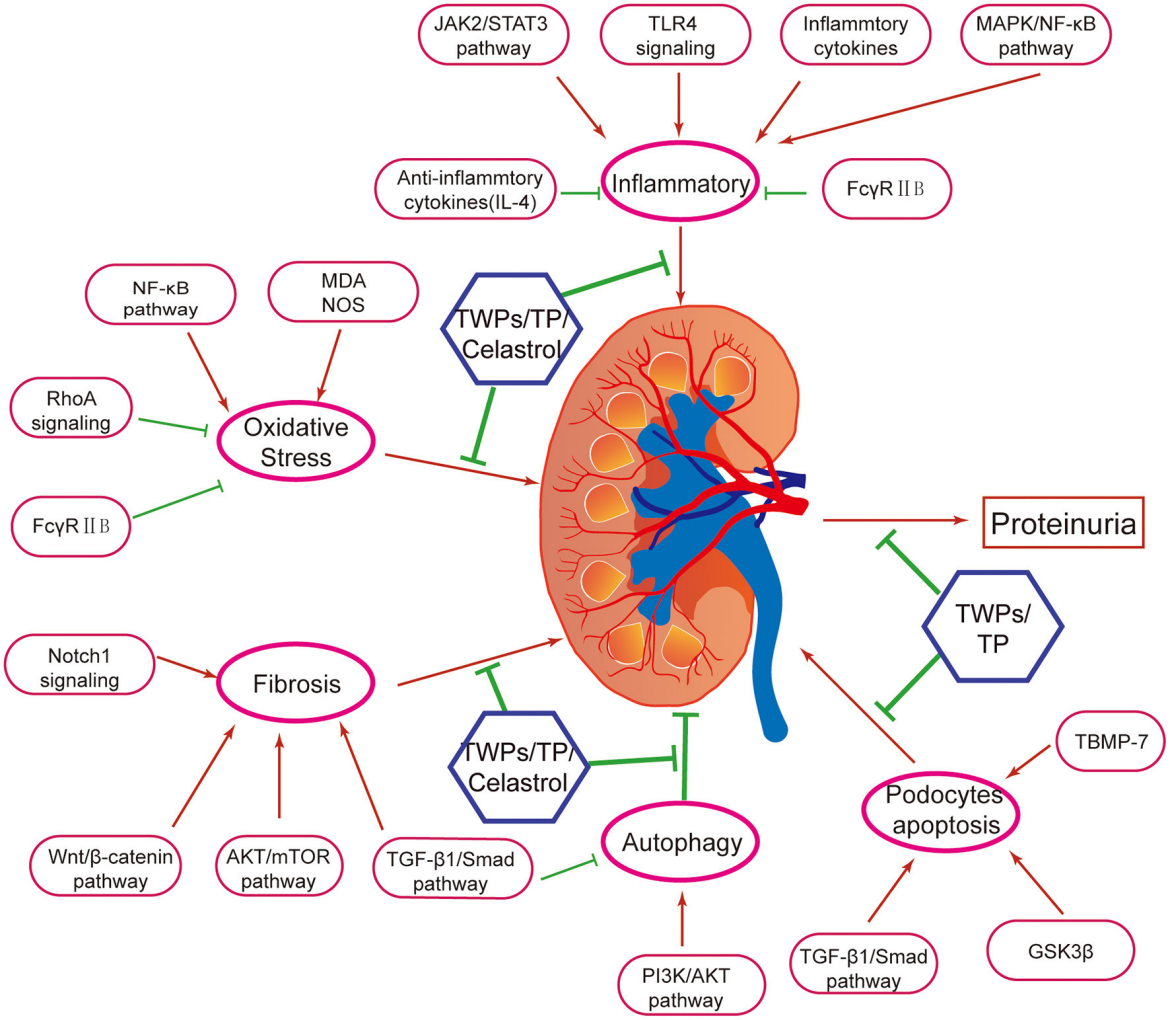
Mechanisms of *Tripterygium wilfordii Hook. f*. and active compounds against proteinuria and kidney injury in DN. TWPs, TP, and Celastrol are the effective medicine against proteinuria and kidney injury in DN. Mechanisms of TWHF, TWPs, TP, and Celastrol are including anti-inflammation, antioxidation, anti-fibrosis, activating autophagy, and anti- podocyte apoptosis, via several mechanisms.

**Figure 3:**
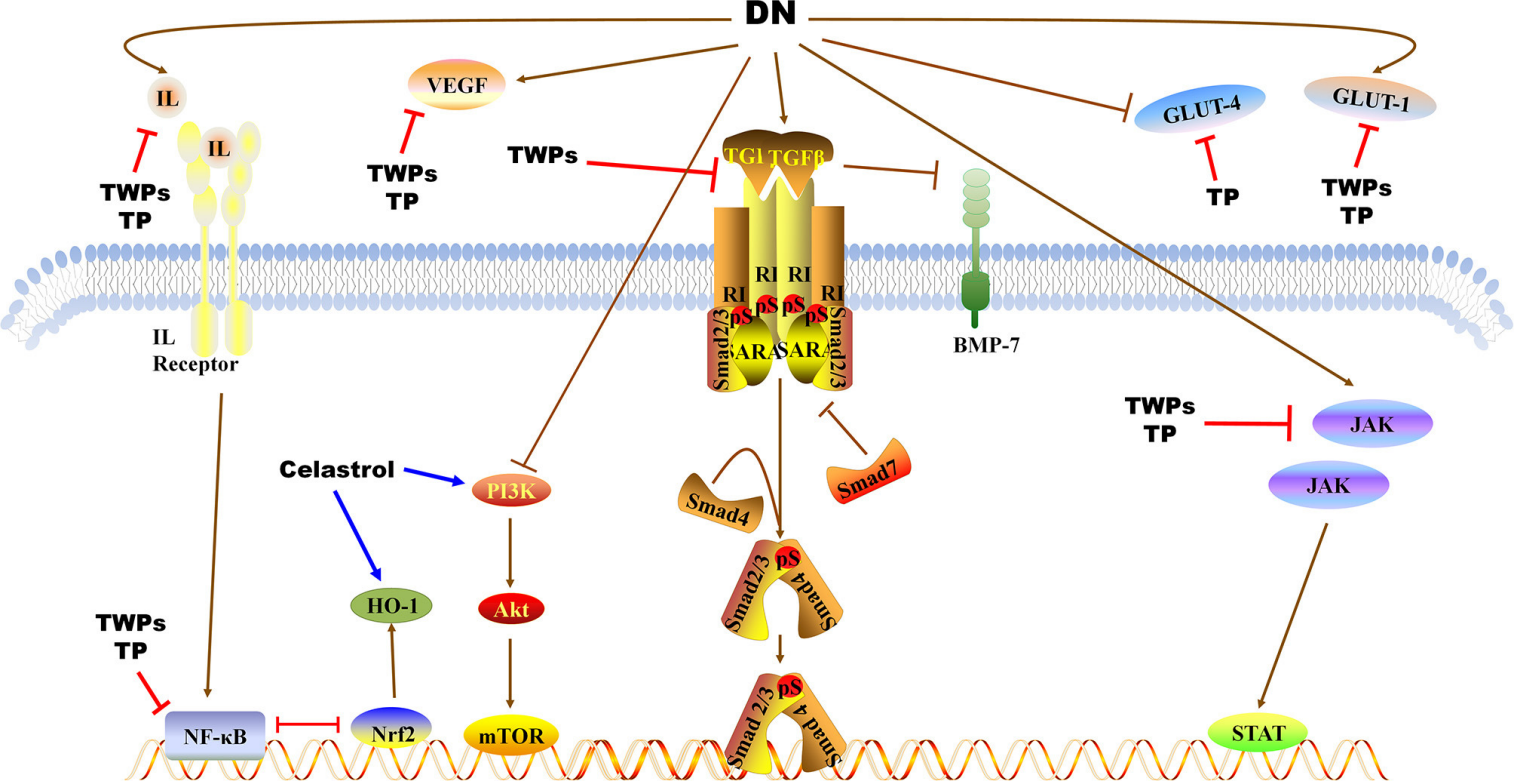
Pathways of TWPs, TP, and Celastrol against proteinuria and kidney injury in DN. TWPs and TP attenuate proteinuria in DN by regulating JAK/STAT pathway, TGF-β1/Smad pathway and NF-κB pathway, and regulating the expressions of IL, VEGF, BMP-7, GLU-1, and GLU-4. Celastrol attenuates proteinuria in DN by regulating PI3K/AKT/mTOR pathway and regulating the expressions of HO-1.

TWPs, triptolide (TP) and celastrol, predominantly active natural products isolated from TWHF, are mainly used to treat DN (Figure 1). As the fat-soluble mixture extracted from the root of TWHF, TWPs are the first CHM studied and used in anti-inflammatory and immune regulation (28). In 1972, Kupchan et al. first isolated and characterized TP from TWHF (26). Celastrol was first isolated from TWHF for the activator of the mammalian heat shock transcription factor 1 (29). The pharmacological activities and mechanisms of TWHF and its active compounds have been extensively investigated in many kidney disease models (Table 1, Figures 2, 3).

**Table 1:** Pharmacological activities of *Tripterygium wilfordii Hook. f*. and active compounds against proteinuria and kidney injury in DN.

**Natural product**	**Underlying mechanisms**	**Model**	**Experimental detail**	**Underlying targets**	**References**
TWPs	Anti-inflammatory	STZ-induced DN male SD rats	9 and 18 mg/kg by gavage for 8 weeks	Reducing serum IL-1, IL-17, IFN-γ levels	(30)
		High-sugar and high-fat diet and STZ-induced DN male SD rats	6, 12, and 24 mg/kg by gavage for 4 weeks	Reducing renal TNF-α expressions, increasing renal IL-4 expressions	(31)
		High-sugar and high-fat diet and STZ-induced DN male SD rats	8 mg/kg by gavage for 8 weeks	Inhibiting the activity of JAK/STAT pathway	(32)
		STZ-induced DN male SD rats	8 mg/kg by gavage for 4 weeks	Inhibiting the activity of MAPK/NF-κB pathway	(33)
		Fetal Bovine serum albumin induced chronic glomerulonephritis Wistar rats	15 mg/kg by gavage for 4 weeks	Inhibiting the activity of p38MAPK pathway	(34)
		Fetal bovine serum albumin to stimulate activated macrophages induced IgAN Wistar rats	20 mg/kg by gavage for 4 weeks	Reducing serum IL-1β, IL-6 levels	(35)
	Antioxidative stress	STZ-induced DN male SD rats	4.5, 9, and 18 mg/kg by gavage for 8 weeks	Reducing renal MDA expressions, increasing renal GPxs expressions	(36)
	Anti- fibrosis	High-sugar and high-fat diet and STZ-induced DN male SD rats	50 mg/kg by gavage for 16 weeks	Reducing renal TGF-β1 and gremlin expressions, increasing renal BMP-7 expressions	(37)
		Male db/db mice	25, 50, and 100 mg/kg by gavage for 8 weeks	Promoting AKT/mTOR pathway	(38)
		STZ-induced DN male SD rats	50 mg/kg by gavage for 8 weeks	Inhibiting renal RhoA and Rock1 expressions	(39)
		Unilateral ureteral obstruction SD rats	10 mg/kg by gavage for 14 days	Inhibiting renal miR-192 and collagen I expressions	(40)
	Anti- podocyte apoptosis	High-sugar and high-fat diet and STZ-induced DN male SD rats	1, 3, and 6 mg/kg by gavage for 8 weeks	Reducing renal VEGF expressions, increasing renal nephrin and podocin expressions	(41)
		Adriamycin- induced nephropathy male SD rats	50 mg/kg by gavage for 8 weeks	Increasing renal nephrin and CD2AP expressions	(42)
		Sunitinib-induced podocytes	40 ng/ml for 48 h	Increasing celluer nephrin and CD2AP expressions	(43)
TP	Anti-inflammatory	High-sugar and high-fat diet and STZ-induced DN male Wistar rats	100 μg/kg by gavage for 8 weeks	Inhibiting of inflammation and macrophage infiltration	(44)
		Cationic bovine serum albumin induced MN male SD rats	200 μg/kg by gavage for 4 weeks	Inhibiting NF-κB Signaling Pathway	(20)
		Fetal bovine serum albumin to stimulate activated macrophages induced IgAN male Wistar rats	200 μg/kg by gavage for 16 weeks	Reducing serum TNF-α, IL-17A, IFN-γ, and IL-4 levels, inhibiting renal NLRP3, and TLR4 expressions	(45)
		Bovine gamma globulin induced IgAN male SD rats	100 and 200 μg/kg by gavage for 8 weeks	Reducing serum IL-1β and IL-18 levels, inhibiting renal IL-1β, Case-1, IL-18, and NLRP3 expressions	(46)
		Female MRL/lpr lupus mice	125 μg/kg by gavage for 9 weeks	Inhibiting renal JAK1/STAT1 Pathway	(47)
LLDT-8 (a TP derivative)		Female MRL/lpr lupus mice	125 μg/kg/2 d by gavage for 9 weeks	Reducing renal IFN-γ, IL-17, IL-6, and TNF-α expressions	(48)
		Murine anti-glomerular basement membrane (GBM) glomerulonephritis male NZW parental mice	125 μg/kg/2 d by gavage for 14 days	Promoting renal Fcγ receptor signaling	(49)
TP	Antioxidative stress	High-sugar and high-fat diet and STZ-induced DN male SD rats	200 μg/kg by gavage for 8 weeks	Reducing renal COX-2 and iNOS expressions	(50)
		STZ-induced DN male SD rats	200 μg/kg by gavage for 4 weeks and 8 weeks	Reducing renal NF-κB, iNOS, eNOS, and VEGF expressions	(51)
		Puromycin aminonucleoside-mediated PAN male SD rats	200 μg/kg by gavage for 21 days	Promoting renal RhoA signaling	(52)
	Anti- fibrosis	High-sugar and high-fat diet and STZ-induced DN male SD rats	100 μg/kg by gavage for 12 weeks	Inhibiting renal miR-137/Notch1 pathway	(19)
		High-fat diet and STZ-induced DN male SD rats	200 μg/kg by gavage for 12 weeks	Inhibiting renal miR-141-3p/PTEN/AKT/ mTOR pathway	(53)
	Activating autophagy	STZ-induced DN male C57BL/6 mice	200 μg/kg by gavage for 12 weeks	Increasing renal Podocin, Bax, and Caspase-3 expressions	(54)
		Puromycin amino nucleotide-cultured mouse podocytes	100 ng/ml for 4 h	Inhibiting renal mTOR pathway	(55)
		aIgA1 from IgAN patients -cultured mouse podocytes	10 ng/ml for 24 h	Inhibiting cellular mTOR pathway	(56)
	Anti- podocyte apoptosis	Glucose and TGFβ1 -cultured mouse podocytes	0.5, 1, and 3 ng/ml for 36 h	Inhibiting phosphorylation of GSK3β	(57)
		Glucose cultured mouse podocytes	8, 16, and 32 ng/ml for 24 h	Increasing cellular nephrin expressions	(58)
		Glucose cultured mouse podocytes	10 ng/ml for 48 h	Increasing cellular synaptopodin and desmin expressions	(59)
		Bovine serum albumin, carbon tetrachloride, and lipopolysaccharide induced IgAN male SD rats	100, 200, and 400 μg/kg by gavage for 4 weeks	Increasing renal nephrin and podocin expressions	(60)
Celastrol	Anti-inflammatory	STZ-induced DN male SD rats	50, 100 μg/kg by gavage for 4 weeks	Inhibiting the activity of MAPK/NF-κB pathway	(33)
		Male db/db mice	1 mg/kg by gavage for 8 weeks	Inhibiting the activity of NF-κB pathway	(61)
	Activating autophagy	High-sugar and high-fat diet and STZ-induced DN male SD rats	1.5 mg/kg by gavage for 4 weeks	Promoting renal PI3K/AKT pathway	(62)
		Glucose cultured mouse podocytes	0.1, 0.2, 0.6, 1.0, 1.5, and 2 μM for 48 h	Promoting cellular HO-1-mediated autophagy	(63)
TWPs	Improving renal hypoxia	STZ-induced DN male SD rats	8, 16 mg/kg, by gavage for 8 weeks	Reducing renal HIF-1α and endothelin-1expressions	(64)
	Improving renal glucose transport	STZ-induced DN male SD rats	1.8 g/kg by gavage for 8 weeks	Reducing renal GLUT-1 expressions	(65)
TP	Improving renal glucose transport	STZ-induced DN male SD rats	1.8 g/kg by gavage for 8 weeks	Reducing renal GLUT-1 expressions, increasing renal GLUT-4 expressions	(66)
TWHF	Anti- fibrosis	STZ-induced DN male SD rats	8 g/kg, and 16 g/kg by gavage for 8 weeks	Inhibiting renal Wnt-1/β-catenin pathway	(67)

## Effects, Mechanisms, and Therapeutic Targets of TWPS Against Proteinuria and Kidney Injury in DN

### Anti-inflammatory Effects

Chronic systemic inflammation is associated with kidney injury, and animal and human studies have established that inflammation is a cornerstone in the development and progression of DN (68, 69). Inflammation can alter or interfere with the regulation and perfusion distribution can induce kidney injury, thereby enhancing the DN progression. Overproduction of Advanced glycation end products (AGEs)or damage from degradation may activate inflammation, which, in turn, promotes DN (70). Thus, the regulation of inflammation is key to the development of treatment schemes for kidney disease.

TWPs exhibit anti-inflammation activity in DN rats. TWPs improve renal inflammatory injury in DN rats by reducing the levels of inflammatory cytokines, such as IL-1, IL-17 and interferon- γ (IFN-γ) (30). TWPs downregulate TNF-α, whereas it upregulated IL-4 (anti-inflammatory T-helper cell type 2 cytokine) in renal tissues (31). The JAK2/STAT3 signaling pathway regulates a broad range of biological effects such as cell proliferation, differentiation, inflammation, and apoptosis (71). Inhibiting JAK2/STAT3 activation, which contributes to the pathogenesis of DN, has been shown to be a novel therapeutic scheme for the treatment of this disease (72). In DN rats, TWPs reduce the levels of BUN, Scr and improve kidney function, and also effectively blank the inflammatory response by inhibiting the activity of JAK/STAT pathway (32). Treatment with TWPs also inhibit inflammation via regulating the signal pathway of MAPK/NF-κB in renal tissues (33).

In bovine serum albumin induced chronic glomerulonephritis rat model, TWPs inhibit the inflammatory factor (TNF-α, IL-1β) expressions, and improve the renal pathological damage via regulating MAPK signaling pathway (34). In immunoglobulin A nephropathy (IgAN) rats, TWPs decrease the levels of serum IL-1β, IL-6, and reduce the pathological damage of renal tissue (35) (Table 1, Figures 2, 3).

### Antioxidative Stress Effects of TWPs

Oxidative stress is associated with inflammation in DN progression. The presence and severity of systemic inflammation contribute to kidney injury-related oxidative stress (73). Oxidative stress caused by the overaccumulation of reactive oxygen species (ROS) induces protein and nucleic acid damage, thereby leading to impaired cellular damage and tissue pathology (74). The mitochondria are the major sources of ROS as well as the main targets of ROS (75). The damaged mitochondria with impaired respiration block the transfer of electrons along the respiratory chain, which then react with O_2_ in upstream respiratory chain components to form superoxide free radicals and ROS (76). In response to the excessive production of ROS, mammalian cells have evolved various peroxidases that catalyze the conversion of intracellular hydrogen peroxide to water. These include catalase, peroxiredoxins, and glutathione peroxidases (GPxs) (77). There is increasing evidence that oxidative stress contributes to DN progression (78, 79). TWPs up-regulate the levels of catalase in serum and GPxs in kidneys, and down-regulated the levels of malondialdehyde (MDA) in kidneys in the DN (36) (Table 1, Figures 2, 3).

### Anti-fibrosis Effects

Renal fibrosis is a highly complex process involving a variety of cell types including resident renal cells as well as infiltrating cells, such as macrophages, fibrocytes, and lymphocytes. Intracellular ROS generation in the context of diabetes initiates multiple inflammatory and profibrotic responses (80). Renal fibrosis in DN is caused by the accumulation of extracellular matrix (ECM) proteins, including predominantly various collagens, fibronectin, and laminin (81). Thickening of the glomerular basement membrane (GBM) is an early histopathological finding in DN (82). Altered GBM remnants contribute to the expansion of the mesangial matrix, but hyperglycemia also stimulates mesangial cells to proliferate and produce matrix by activating transforming growth factor-β (TGF-β) and vascular endothelial growth factor (VEGF), which directly induce the transcriptional activation of matrix collagens (83). It is currently believed that renal fibrosis develops in response to ECM accumulation due to epithelial-mesenchymal transition (EMT), TGF-β signaling, oxidative stress and proteinuria (84, 85).

TGF-β1/Smad signaling pathway plays a critical role in prolonged glomerulosclerosis, which is an important determinant during the progression in DN (86). Bone morphogenetic protein-7 (BMP-7) is a critical developmental and differentiation factor in the kidney, which can inhibit TGF-β signaling to ameliorate renal inflammation, apoptosis, and fibrosis after kidney injury (87, 88). In DN rats, TWPs ameliorate renal fibrosis by down-regulating the expression of TGF-β1 and gremlin (a BMP antagonist), and up-regulating the expression of BMP-7 (37). In db/db mice, TWPs reduce the serum levels of TC, TG, and LDL, glycated serum protein, BUN, Scr, and improve the renal injury by regulating AKT/mTOR pathway (38). And TWPs inhibit the expressions of RhoA and Rock1 to improve renal fibrosis in STZ-induced rats (39).

MicroRNAs (miRNAs) are a class of small non-coding RNAs that regulate gene expression by either downregulating mRNA levels or directly repressing translation of genes. Many miRNAs are corrected with renal injury in DN (89, 90). In unilateral ureteral obstruction rats, TWPs could attenuate renal fibrosis by inhibiting the expression of miR-192 and collagen I (40) (Table 1, Figures 2, 3).

### Anti-podocyte Apoptosis Effects

Podocyte injury is a pathological feature in DN. Podocytes are highly specialized, terminally differentiated epithelial cells in the glomerular filtration barrier with interdigitating foot processes (FPs), and play a major role in preventing protein leakage into the Bowman space (91). Structural podocyte injury is central in the pathogenesis of most inherited and acquired glomerular diseases, which are all associated with decreased expression of slit diaphragm (SD) proteins, such as podocin, nephrin, synaptopodin, and CD2-associated protein (CD2AP) (92). These proteins are considered as critical components of epithelial SD and FPs and help maintain the integrity of podocytes in avoiding proteinuria (93). In addition, desmin is a component of the cytoskeleton and considered as a sensitive marker of injury in podocytes (94). DM induces podocytopathy, which is characterized by cellular hypertrophy, foot process effacement, and podocyte loss (6). Li et al. (41) showed using STZ-induced DN rats that TWPs could upregulate the expression of nephrin and podocin and suppress apoptosis in podocytes.

TWPs have also been shown to significantly reduce proteinuria and repair podocyte damage in rats with adriamycin-induced nephropathy, as well as facilitate mixing together of foot processes by upregulating nephrin and CD2AP (42). In addition, TWPs upregulates nephrin and CD2AP in sunitinib-induced podocytes (43) (Table 1, Figures 2, 3).

## Effects, Mechanisms, and Therapeutic Targets of TP Against Proteinuria and Kidney Injury in DN

### Anti-inflammatory Effects

Due to similar structures as hormones, TP can bind to nuclear receptors (95). This unique feature is the reason that triptolide is active to inflammation. Ma et al. (44) have shown that TP markedly attenuated proteinuria and renal injury in DN rats, which may have been correlated with the inhibition of macrophage infiltration and inflammation in the kidneys.

Chronic inflammation is also a common characteristic of membranous nephropathy (MN) and IgAN. Zhou et al. (20) concluded that TP significantly reduces the production of inflammatory cytokines (e.g., IL-1β, TNF-α, and monocyte chemotactic protein 1), and inhibits the NF-κB signaling pathway in MN rats. He et al. (45) declared that TP prevents IgAN progression via by ameliorating of inflammasome-mediated proinflammatory cytokine production by down-regulating Toll-like receptor 4 (TLR4) and nod-like receptor family pyrin domain-containing 3 (NLRP3) expression. In IgAN rats, TP decrease the levels of TNF-α, IL-17A, IFN-γ, and IL-4 in serum, reduce the expression of IL-1β, Caspase-1, IL-18, and NLRP3 in renal tissues (46).

In MRL/lpr lupus mice, TP also inhibition of inflammatory response, ameliorate renal damage, and the mediated by JAK1/STAT1 pathway is a possible molecular mechanism (47).

Zhang et al. (48) have shown that (5R)-5-hydroxytriptolide (LLDT-8, a TP derivative) provides therapeutic benefits to LN by suppressing chemokine expression and inhibiting immune cell infiltration in the kidneys of MRL/lpr mice. Moreover, LLDT-8 inhibits inflammation in the kidneys by downregulating the cytokines IL-6, IL-17, TNF-α, and IFN-γ and upregulating FcγRIIB in the kidneys of a murine anti-glomerular basement membrane (GBM) glomerulonephritis model (49) (Table 1, Figures 2, 3).

### Antioxidative Stress Effects

TP effectively attenuates the levels of blood glucose, Scr and proteinuria by reducing the expression of cyclooxygenase-2 (COX-2) and inducible nitric oxide synthase (iNOS) in renal tissues of DN rats (50). NF-κB is a redox-sensitive transcription factor that responds to ROS at various sites within the signaling pathway such as by activating or inactivating the inhibitory κB kinase complex, which, in turn, affects downstream targets or activates NF-κB via alternative inhibitor κBα phosphorylation (96). TP protects glomerular endotheliocytes of DN by inhibiting the expression of NF-κB, iNOS, endothelial nitric oxide synthase (eNOS), and VEGF (51).

RhoA, a redox sensitive master regulator protein, regulates numerous biological functions (97). Due to lipid peroxidation is a major form of oxidative stress in diabetes, restoring normal RhoA activity levels prevents podocyte loss and consequent proteinuria in DN (98). Zheng et al. (52)concluded that TP ameliorated puromycin amino nucleoside-mediated podocyte injury by suppressing ROS generation and p38 mitogen-activated protein kinase activation while restoring RhoA signaling activity *in vivo* and *in vitro* (Table 1, Figures 2, 3).

### Anti-fibrosis Effects

The Notch1 signaling plays a core role in the formation of mesangial cells during kidney development, and exacerbates renal tubulointerstitial fibrosis in DN (99). Han et al. (19) declared that TP has anti-glomerulosclerosis effects by suppressing miR-137/Notch1 pathway in DN rats. In addition, renal fibrosis can be regulated through autophagy, a biological regulatory program that maintains homeostasis (100). Phosphatase and tensin homolog deleted on chromosome ten (PTEN) plays an essential role in regulating of AKT/ mammalian target of rapamycin (mTOR) signaling (101). Li et al. (53) found that TP alleviates renal fibrosis by restoring autophagy through the miR-141-3p/PTEN/AKT/mTOR pathway in DN rats (Table 1, Figures 2, 3).

### Autophagy Regulatory Effects

Autophagy is a highly conserved and lysosome-dependent bulk degradative pathway that participates in the clearance of damaged organelles and proteins, as well as in maintaining homeostasis in tubules and glomeruli (102). Deficiency in autophagy aggravates DN in rodent models. STZ-induced autophagy-deficient mice develop severe microalbuminuria, endothelial lesions, and podocyte damage (103). High-fat diet-induced podocyte-specific autophagy-deficient mice develop hyperglycemia with proteinuria and podocyte damage. Autophagy contributes to the degradation of AGEs and suppresses inflammation in the kidneys (104). Moreover, increased ROS enhances autophagy by controlling the activity of Atg4, a family of cysteine proteases that is essential for autophagy formation (105). ROS promotes autophagy through the activation of AMP-activated protein kinase (AMPK), likely via suppression of mTOR (106). Experimental evidence has shown that autophagy acts as a double-edged sword with regard to cell death and survival because it is accompanied by other forms of cell death such as apoptosis (107). The ratio of LC3 I to LC3 II is closely correlated with the extent of autophagosome formation; therefore, LC3 II could be a marker of autophagic activity (108). In STZ-induced rats, TP decrease the expression of LC3 II, inhibite autophagy by upregulating PI3K/Akt/mTOR pathway (54). In puromycin amino nucleotide-cultured podocytes, TP reduces podocyte injury via the mTOR-autophagy pathway to increase autophagy levels and facilitates podocyte recovery from injury (55). Autophagy may be regulated by mTOR complex 1 (mTORC1) (109). Haploinsufficiency of mTORC1 in podocytes or administration of rapamycin (a mTORC1 inhibitor), resulting in the activation of autophagy, has been shown to prevent progressive DN (106). Conversely, the activation of mTORC1 in podocytes, which results in the inhibition of autophagy, leads to accelerated DN (110). Furthermore, Liang et al. found that TP protects podocyte autophagy by suppressing the mTOR and AKT pathways in IgAN (56) (Table 1, Figures 2, 3).

### Anti-podocyte Apoptosis Effects

In glucose and TGFβ1-cultured mouse podocytes, TP protected podocytes against diabetic milieu-elicited injury, mitigated cytoskeleton derangement, and preserved podocyte filtration barrier function via inhibiting phosphorylation of GSK3β (57). In glucose-cultured mouse podocytes, TP increases renal synaptopodin, desmin, and nephrin expressions to ameliorate podocyte injury (58, 59). Similarly, TP could significantly decrease proteinuria and upregulate nephrin and podocin mRNA and protein expression in rats with IgAN, suggesting that TP could reduce podocyte injury and repair glomerular filtration membrane barrier damage (60) (Table 1, Figures 2, 3).

## Effects, Mechanisms, and Therapeutic Targets of Celastrol Against Proteinuria and Kidney Injury in DN

### Anti-inflammatory Effects

As one of triterpenes in TWHF, Celastrol reduces levels of Scr, BUN and proteinuria, inhibits inflammation by regulating MAPK/NF-κB pathway in STZ-induced rats (33). In db/db mice, Celastrol improves insulin resistance and attenuates renal injury by inhibiting the NF-κB-mediated inflammatory (61) (Table 1, Figures 2, 3).

### Autophagy Regulatory Effects

The PI3K/AKT pathway is one of the most important signaling pathways that regulate autophagy, and phosphorylated AKT can promote the formation of p-mTOR to inhibit cell autophagy (111). In STZ-induced rats, Celastrol attenuates renal injury by promoting the PI3K/AKT pathway to activate autophagy (62). As a proverbial cytoprotective enzyme, heme oxygenase-1 (HO-1) ameliorates cell injury and inflammation in podocytes via activating autophagy pathway. Celastrol protects against high glucose-induced podocyte injury by restoring HO-1-mediated autophagy pathway (63) (Table 1, Figures 2, 3).

## Other Effects of TWHF and Its Main Bioactive Ingredients

Glomerular hypertension and tubulointerstitial hypoxia occur following DN, causing loss of glomerular integrity and tubular damage (112). Hypoxia inducible factor 1 α (HIF-1α) plays a regulatory role in cellular response to renal hypoxia. Chen et al. (64) drew a conclusion that TWPs decreased levels of Scr, BUN, 24-h UAlb, mean glomerular area and mean glomerular volume; improved renal histopathology; and down-regulated the expression of HIF-1α and endothelin-1 mRNA and protein in the kidneys of diabetic rats. HIF-1α activation under hypoxia could upregulate downstream glucose transporter 1 (GLUT-1) gene (113). TWPs and TP significantly reduce proteinuria and GLUT-1 levels in glomerular mesangial and epithelial cells of DN rats (65, 66).

Wnt/β-catenin signaling is an evolutionary conserved signaling pathway, which plays a core role in modulating kidney injury and repair (114). In DN rats, Chang et al. drew a conclusion that TWHF mitigates hyperglycemia-induced upregulated Wnt-1 and β-catenin expression in kidney tissues and ameliorates kidney injury (67) (Table 1, Figures 2, 3).

## Conclusions

In this review, we have summarized currently available information on the effects of TWHF on DN. Experimental studies have demonstrated that TWHF interacts with a wide range of cellular processes such as inflammation, oxidative stress, fibrosis, apoptosis, autophagy, and podocytes, indicating that these mechanisms are involved in a variety of cellular signals. Although several genes and proteins involved in the effect of TWHF on cells and tissues have been identified, many of the targets and exact mechanisms participating in these events remain unknown. Further studies regarding the mechanism of DN with TWHF treatment are thus warranted. Its narrow therapeutic window and severe side effects restrict its clinical applications (26, 27). Therefore, hepatotoxicity and sexual inhibition may occur among patients who have used TWHF long term, thus requiring regular monitoring, and if necessary, a reduction in dose or possibly termination of its use.

## Author Contributions

PL, JZ, D-QC, and XQ mainly drafted the work critical for important intellectual content. YW, ZS, and CW finished the discussion. PL and JZ contributed equally to this work. All authors contributed to the article and approved the submitted version.

## Funding

This work was supported by supported by Research Projects of the National Natural Science Foundation of China (No. 81904174), China Postdoctoral Science Foundation (No. 2021M693579), and National Training Program for Innovative Key Talents of Traditional Chinese Medicine (No. 2019-128).

## Conflict of Interest

The authors declare that the research was conducted in the absence of any commercial or financial relationships that could be construed as a potential conflict of interest.

## Publisher's Note

All claims expressed in this article are solely those of the authors and do not necessarily represent those of their affiliated organizations, or those of the publisher, the editors and the reviewers. Any product that may be evaluated in this article, or claim that may be made by its manufacturer, is not guaranteed or endorsed by the publisher.
